# The complete chloroplast genome sequence of *Duranta erecta* (Verbenaceae)

**DOI:** 10.1080/23802359.2021.1934164

**Published:** 2021-06-03

**Authors:** Wei-cai Song, Qi Feng, Yun-jiao Zhang, Xiao-meng Wu, Chao Shi, Shuo Wang

**Affiliations:** a College of Marine Science and Biological Engineering, Qingdao University of Science and Technology, Qingdao, China; b Kunming Medical University Haiyuan College, Kunming, China

**Keywords:** Verbenaceae, *Duranta erecta*, chloroplast genome, phylogenetic relationship

## Abstract

In this study, the complete chloroplast (cp) genome of *Duranta erecta* was assembled using Illumina sequencing data. The complete cp genome is 149,869 bp in length, including a pair of invert repeats (IRA and IRB) regions of 22,839 bp, large single-copy (LSC) region of 86,201 bp, and small single-copy (SSC) region of 17,990 bp. The G + C content of this cp genome was 38.26%. A total of 128 genes were predicted in the genome, including 83 protein-coding genes, 37 tRNA genes, and eight rRNA genes. Phylogenetic analysis confirmed the phylogenetic relationship between *D. erecta* and other representative species of Verbenaceae.

*Duranta erecta*, commonly called ‘Golden Dew Drops’, belongs to the family Verbenaceae (Aymard and Grande 2012). *Duranta erecta* is the most variable and commonly cultivated species of the genus (Moroni et al. 2018). It is an erect shrub, about 1–3 m in height, with prickly branches and pilose young shoots; leaf blade is ovate-elliptic or ovate-lanceolate, 2–6.5 cm long, 1.5–3.5 cm wide; the flowers are often involucres of colored bract, with yellow or yellow-orange fruits at maturity, about 5–13 mm in diameter, and grow in hanging clusters (Said 2016). It is popular as a wild ornamental plant and mainly grows in arid coastal areas with elevations ranging from near sea level to more than 100 m (Dew et al. 2020). At the same time, as an important source of natural medicine, it is still widely used to treat a variety of diseases (Puri 2018). Previous studies have shown that it has been used to treat toothaches, headaches, protect the liver, heal wounds and as a diuretic (Ugwu and Anosike 2020). Its fruit is also used to treat malaria and intestinal worms (Nikkon et al. 2008). In this paper, we report the chloroplast (cp) genome of *D. erecta* at the first time, which provides important information for further taxonomic and population genetics studies of the species (Ahmed et al. 2012; Guo et al. 2017).

Fresh leaves of *D. erecta* were collected from Kunming city, Yunnan province of China (24°23′N, 102°10′E), and the voucher specimen and DNA were deposited at Qingdao University of Science and Technology (specimen code HY0874). The total genomic DNA was extracted from fresh leaves of the sample using a modified CTAB method (Doyle and Doyle 1987). A genomic library with an insert size of 450 bp was constructed and the library was sequenced using the Illumina HiSeq platform in Novogene (Nanjing, China). A total of 3 Gb of 150 bp paired-end reads were generated. We also deposited the raw sequencing reads in SRA with accession no. SRR13825327.

The complete cp genome sequence of *D. erecta* was assembled using the program NOVOPlasty4.2 software (Dierckxsens et al. 2017), with ribulose-1,5-bisphosphate carboxylase/oxygenase (rbcL) gene from Duranta erecta voucher OP9 (GenBank accession no. JX887620) as the seed sequence. The final complete cp genome annotation was performed using the GeSeq (Michael et al. 2017) and tRNAs were identified using the tRNAscan-SE v2.0.7 (Schattner et al. 2005).

The complete cp genome of *D. erecta* (GenBank accession no. MW525381) was 149,869 bp in length, with a GC content of 38.26%. The complete cp genome shows a typical quadripartite structure with a pair of inverted repeats (IRA and IRB) regions of 22,839 bp, large single-copy (LSC) region of 86,201 bp, and small single-copy (SSC) region of 17,990 bp. A total of 128 genes were identified from *D. erecta* cp genome, including 83 protein-coding genes, 37 tRNA genes, and eight rRNA genes, eight classes of genes (rps16, atpF, petB, petD, rpl16, ndhB, ndhA, rpl2) were found to contain two exons.

The complete cp genome sequence of *D. erecta* and other species selected from the Verbenaceae were used to construct phylogenetic tree. *Catalpa ovata* was selected as the outgroup of the phylogenetic tree (Wortley et al. 2005). The sequences were downloaded from the GenBank database and were aligned using MAFFT version 7 (Katoh and Standley 2013) and then visualized and manually adjusted using BioEdit (Hall 1999). Model selected process in Mega version X (Kumar et al. 2018). GTR + G+I were selected by the Akaike information criterion. The maximum-likelihood (ML) method was used to infer the phylogenetic relationship, and the phylogenetic tree was constructed with Mega version X using 1000 bootstrap (Minh et al. 2013). The result phylogenetic tree shows that *D. erecta* was clustered with other species from genus *Lantana camara* (Figure 1). This result defines the phylogenetic position of *D. erecta* at molecular level in Verbenaceae for the first time, and provides a theoretical basis for its molecular identification and resource development and utilization.

**Figure 1.**
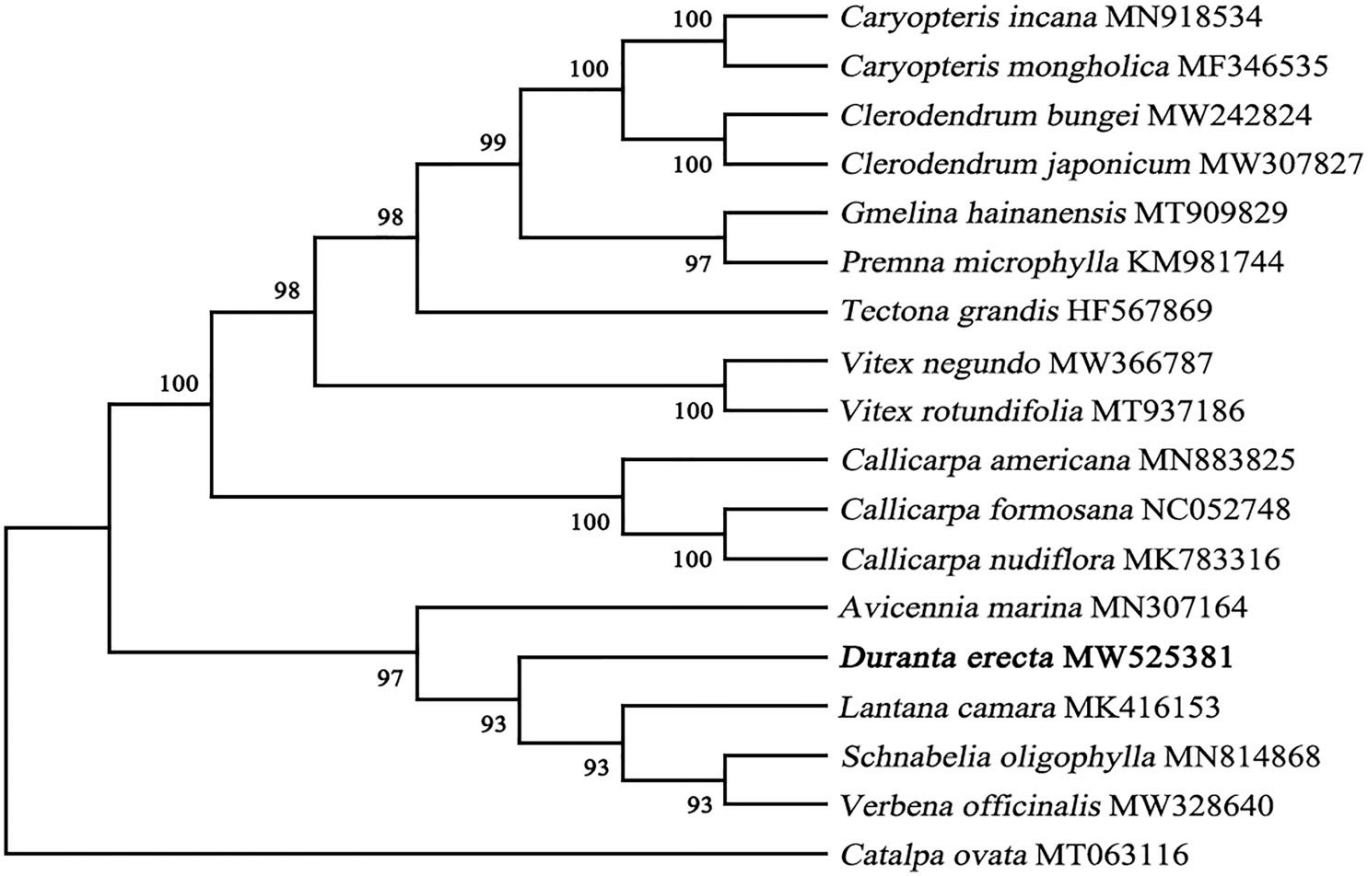
A maximum-likelihood (ML) tree illustrates the phylogenetic position of *D. erecta* among part of Verbenaceae species. The number on each node indicates bootstrap support value. After species is the chloroplast genome sequence login number used by GenBank.

## Data Availability

The genome sequence data that support the findings of this study are openly available in GenBank of NCBI at https://www.ncbi.nlm.nih.gov/ under the accession no. MW525381. The associated BioProject, SRA, and Bio-Sample numbers are PRJNA705870, SRR13825327, and SAMN18105487, respectively.
